# Computational Study on DNA Repair: The Roles of Electrostatic Interactions Between Uracil-DNA Glycosylase (UDG) and DNA

**DOI:** 10.3389/fmolb.2021.718587

**Published:** 2021-08-06

**Authors:** Yixin Xie, Chitra B. Karki, Jiawei Chen, Dongfang Liu, Lin Li

**Affiliations:** ^1^ Computational Science Program, University of Texas at El Paso, El Paso, TX, United States; ^2^ Computer Science Program, Santa Monica College, Santa Monica, CA, United States; ^3^ Department of Computer Engineering, Rochester Institute of Technology, Rochester, NY, United States; ^4^ Department of Physics, University of Texas at El Paso, El Paso, TX, United States

**Keywords:** uracil-DNA glycosylase, UDG enzyme, DNA damage, DNA repair, base excision repair, folding energy, electrostatic force, electric field line

## Abstract

Uracil-DNA glycosylase (UDG) is one of the most important base excision repair (BER) enzymes involved in the repair of uracil-induced DNA lesion by removing uracil from the damaged DNA. Uracil in DNA may occur due to cytosine deamination or deoxy uridine monophosphate (dUMP) residue misincorporation during DNA synthesis. Medical evidences show that an abnormal expression of UDG is related to different types of cancer, including colorectal cancer, lung cancer, and liver cancer. Therefore, the research of UDG is crucial in cancer treatment and prevention as well as other clinical activities. Here we applied multiple computational methods to study UDG in several perspectives: Understanding the stability of the UDG enzyme in different pH conditions; studying the differences in charge distribution between the pocket side and non-pocket side of UDG; analyzing the field line distribution at the interfacial area between UDG and DNA; and performing electrostatic binding force analyses of the special region of UDG (pocket area) and the target DNA base (uracil) as well as investigating the charged residues on the UDG binding pocket and binding interface. Our results show that the whole UDG binding interface, and not the UDG binding pocket area alone, provides the binding attractive force to the damaged DNA at the uracil base.

## Introduction

DNA damage happens with a rate of ten thousand to one million molecular lesions per cell every day (Alberts, 2008). It may be caused by endogenous damages, such as reactive oxygen species (ROS), and exogenous damages, such as X-ray and UV radiation, plant toxins, and viruses (Jackson and Bartek, 2009). To keep cells functioning normally, DNA repair is an essential process as it provides comprehensive coverage of cellular responses to DNA damage. Many studies (Acharya, 1972; Capri et al., 2006) have shown that several lifespan-influenced genes turn out to be related to DNA damage repair and protection. DNA repair is an important mechanism that includes base excision repair (BER), nucleotide excision repair (NER), and mismatch repair (MMR) (Wood et al., 2001; Helleday et al., 2008). Among these mechanisms, BER is the process of removing damaged bases which may cause mutations by mispairing or even result in DNA damage (Liu et al., 2007).

Uracil-DNA glycosylase (UDG) is one of the most important enzymes in the BER process (Lindahl, 1993; Schormann et al., 2014). In the DNA duplication process, uracil bases occur due to cytosine deamination or deoxy uridine monophosphate (dUMP) residue misincorporation during DNA synthesis (Longo et al., 1990; Schormann et al., 2014), which leads to a change in the base pair of guanine-cytosine (GC) to adenine-uracil (AU), and over 50% of all the progeny DNA are affected at the mutation site (Pearl, 2000). During the repairing process, UDG detects the damaged DNA base pair AU in a double-stranded DNA by identifying the unusual kink of 45° (Satange et al., 2018). Based on this fact, UDG first scans the DNA backbone for uracil bases, then uses its “pocket” to closely bind to the uracil, and finally catalyzes the hydrolysis of the N-glycosylic bond between uracil and sugar, leaving an apyrimidinic site in the uracil-containing single- or double-stranded DNA (KROKAN et al., 1997). Note that the UDG enzyme shows no activity on uracil of RNA (Parikh et al., 1998).

UDG has been analyzed from variable perspectives for decades (Smith et al., 1993; Sun et al., 1995). Researchers identified UDG in several families (Lee et al., 2011) including Archaea, Eubacteria, Eukaryotes, and large DNA viruses. Many groups studied the consequences of lacking UDG functional activity in human cell lines (Dusseau et al., 2001), which is related to colorectal cancer. Besides, many other investigations were conducted, such as partial UDG treatment for screening of DNA samples (Rohland et al., 2015). Based on various studies, UDG is now widely used in real-time polymerase chain reaction (PCR) to prevent uracil residues in DNA strands (Pierce and Wangh, 2004) and is considered a target for improving the anticancer effects of 5-fluorodeoxyuridine (5-FdU; floxuridine), which is essential in fighting against multiple cancers (Yan et al., 2016). Due to UDG’s crucial functions in many fields, we were motivated to study its detailed mechanisms by using computational methods in biophysics.

Multiscale computational approaches have been widely used to study the protein–protein interactions, which have been proved to be successful (Kortemme et al., 2004; Huang et al., 2013; Li et al., 2016a; Li et al., 2016b). In this study, multiple computational approaches were applied to study the UDG–DNA complex. We calculated the pH dependence of UDG’s folding energy by using DelPhiPKa (Wang et al., 2015a) and electrostatic feature calculations by using DelPhi (Li et al., 2012; Li et al., 2013) and DelPhiForce (Li et al., 2017a). Data analysis and visualization were performed by using Chimera, visual molecular dynamics (VMD) (Humphrey et al., 1996), and R language (with ggplot2 package). First, we calculated the pH dependency of UDG folding energy, and the results show that UDG attains the most stable configuration at pH ranging from 5 to 10. Then, the electrostatic potentials on the surface of both UDG and DNA were calculated, in which different charge distributions of the UDG pocket side and the non-pocket side were analyzed. The calculations of the electrostatic forces between UDG and DNA, especially the pocket area in UDG and the target uracil base in DNA, demonstrate that UDG has overall attractive force to DNA at different distances ranging from 20 Å to 40 Å. Surprisingly, the UDG pocket has repulsive forces to the uracil base at the same distance range. Besides, the residues in both the pocket area and the interfacial area between UDG and DNA was also discussed in detail, which explains the differences of the peculiar force features between the whole UDG binding interface and the UDG binding pocket alone. This research provides the essential explanations of the mechanisms of UDG, i.e., the whole UDG binding interface, and not the UDG pocket area alone, provides the binding attractive force to the damaged DNA. Our findings will shed light on the current UDG enzyme applications and DNA repair mechanisms.

## Methods

### Structure Preparation

**FIGURE 1:**
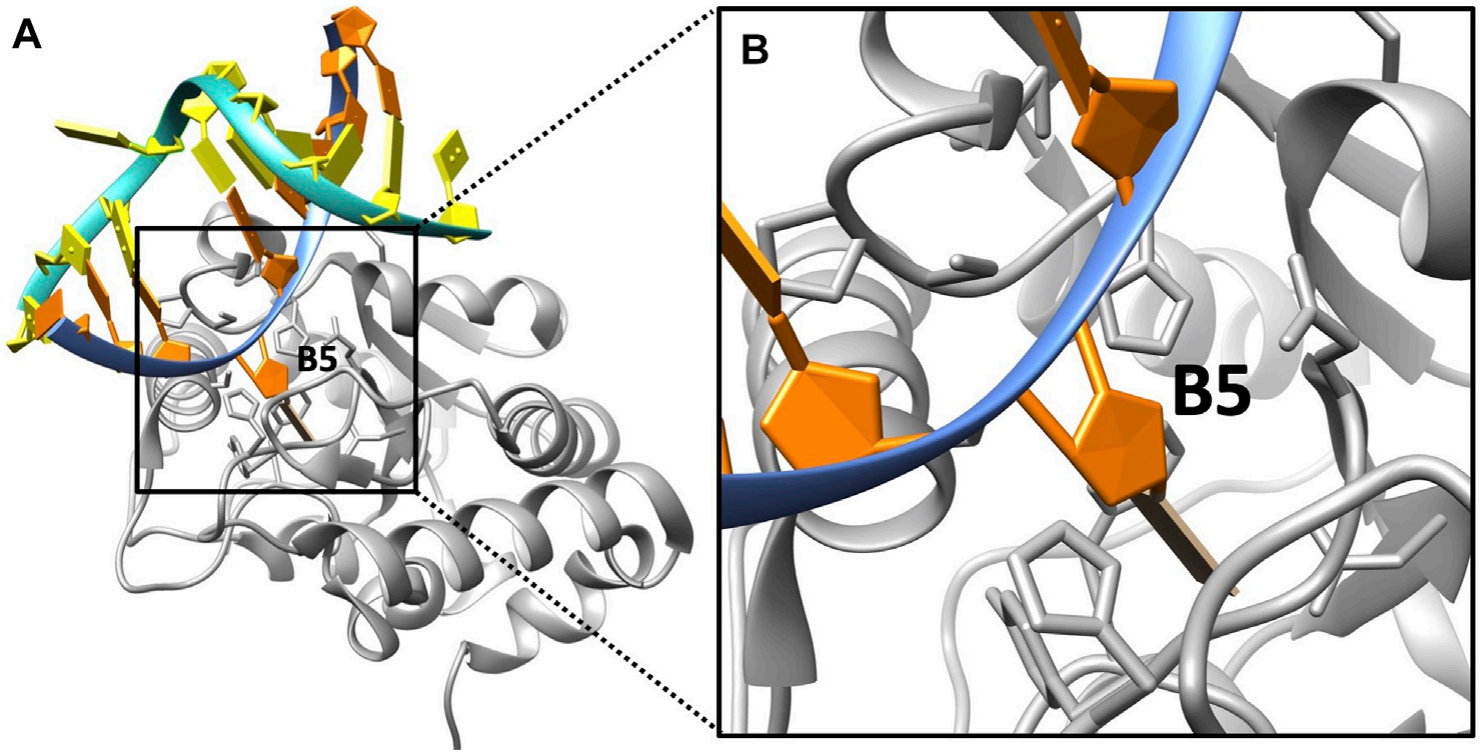
DNA/UDG complex structure. **(A)** The overall structure of UDG (gray) and a double-stranded DNA of which the uracil (at B5 location) flips out to the pocket. **(B)** A zoom-in view of the binding area of UDG (gray) and the uracil (at B5 location).

The complex structure of DNA/UDG was downloaded from the Protein Data Bank (PDB ID: 1EMH (Parikh et al., 2000); Figure 1), and we visualized it by using Chimera (Pettersen et al., 2004). In this original structure, the base at the target location (B5, flips out to UDG) is a pseudosubstrate (P2U), which is to replace uracil so that it binds to UDG stably. Since UDG targets uracil in a real DNA base rather than P2U, we mutated the P2U base to uracil (U) using Chimera. We deleted all the water molecules that are involved in the original structures, as DelPhi (Li et al., 2012), DelPhiForce (Li et al., 2017a), and DelPhiPKa (Wang et al., 2015b) implement an implicit solvent model (Poisson–Boltzmann) in the calculations, which have been proved to be successful in previous studies (Jia et al., 2017; Xie et al., 2020a; Xie et al., 2020b; Guo et al., 2021; Lopez-Hernandez et al., 2021).

As we mentioned previously, in the repairing process of UDG applied on uracil-induced DNA, the incorrect base uracil (U) of DNA (location: B5; Figure 1B) is the target base for UDG to hydrolyze. In order to study the presence of this uracil in a DNA chain and compare it with the original base (before the damage) at this location, which is cytosine (C), we generated a new DNA/UDG structure using cytosine to replace uracil at the B5 position, with the help of Chimera. To better discuss the two structures in the following, we named the DNA with uracil as DNA_RU and the DNA with an original cytosine as DNA_C.

**FIGURE 2:**
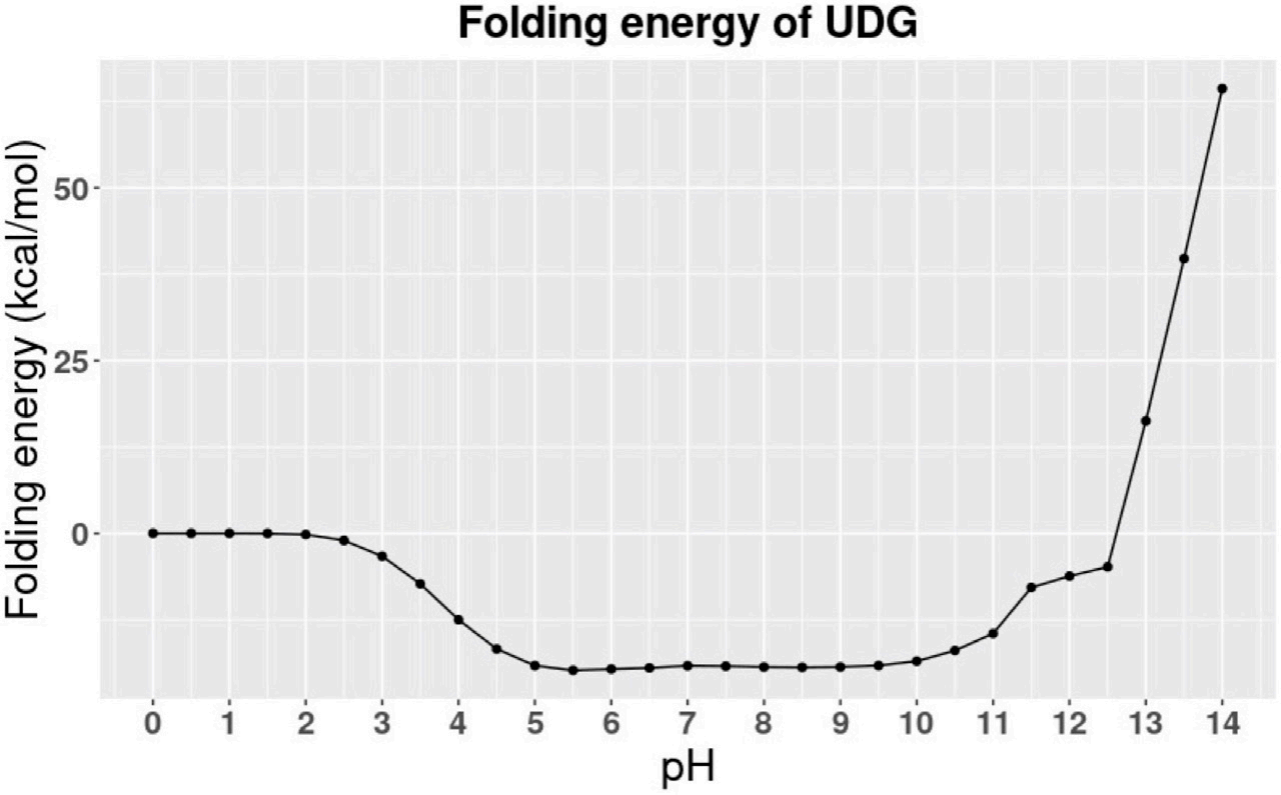
The pH dependence of UDG’s folding energy.

**FIGURE 3:**
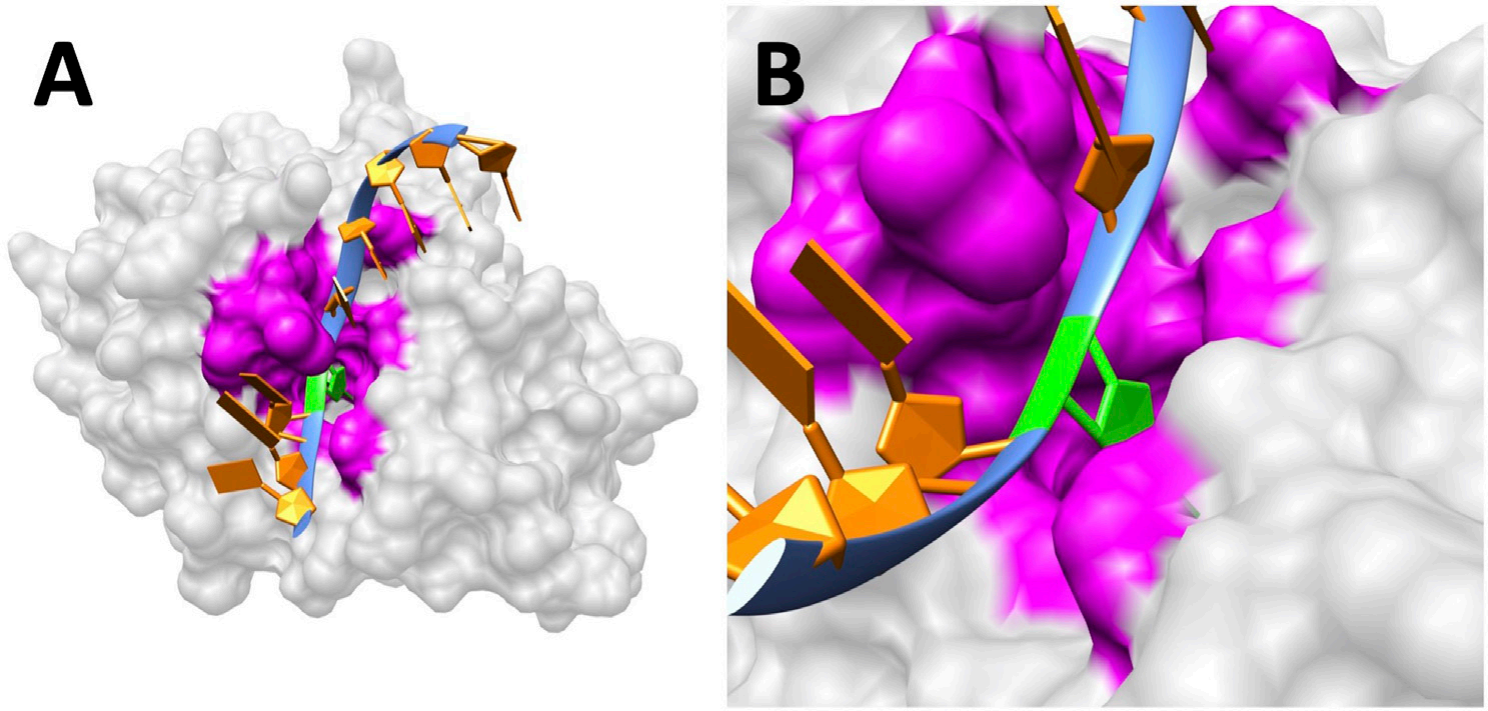
UDG binding pocket. **(A)** The surface of UDG with the pocket colored in magenta. The B5 base uracil (green) flips out to the pocket. **(B)** A closeup of the binding pocket (magenta) surface and the uracil base (green).

The folding energy pH dependency of the entire UDG enzyme is shown in Figure 2, and the detailed discussion is included in Results and Discussions section. In particular, we studied the binding pocket of UDG, which is referred to as the essential binding area (Parikh et al., 1998). The surface of the binding pocket is colored in magenta as shown in Figure 3, and the residues involved in the UDG pocket are Q144, D145, P146, Y147, H148, F158, S169, S247, H268, P269, S270, P271, L272, and S273.

### Electrostatic Potential Calculations

In order to study the electrostatic features, DelPhi (Li et al., 2012; Li et al., 2013) was utilized to calculate the electrostatic potential of DNA and UDG. In the framework of continuum electrostatics, DelPhi calculates the electrostatic potential ϕ (in systems comprised of biological macromolecules and water in the presence of mobile ions) by solving the Poisson–Boltzmann equation (PBE):

$$\begin{array}{c} \nabla \text{·}\left[\epsilon \left(r\right)\nabla \phi \left(r\right)\right]=-4\pi \rho \left(r\right)+\epsilon \left(r\right)\kappa^{2}\left(r\right)\sinh\left(\phi \left(r\right)/k_{B}T\right) \end{array},$$ (1)

where  ϕ(r) is the electrostatic potential, ϵ(r) is the dielectric distribution, ρ(r) is the charge density based on the atomic structures, κ is the Debye–Huckel parameter, $k_{B}$ is the Boltzmann constant, and T is the temperature. Due to the irregular shape of macromolecules, DelPhi uses a finite difference (FD) method to solve the PBE.

Before the DelPhi calculations, the PQR files of DNA and UDG were generated by the PDB2PQR (Dolinsky et al., 2004) tool. We used the AMBER force field for PDB2PQR calculation and removed the water molecules in the process, using the PDB2PQR web server (https://server.poissonboltzmann.org/pdb2pqr). The PDB2PQR built the new hydrogen atoms in proper distances with existing atoms to avoid clashes which also optimized the hydrogen bonding network.

**FIGURE 4:**
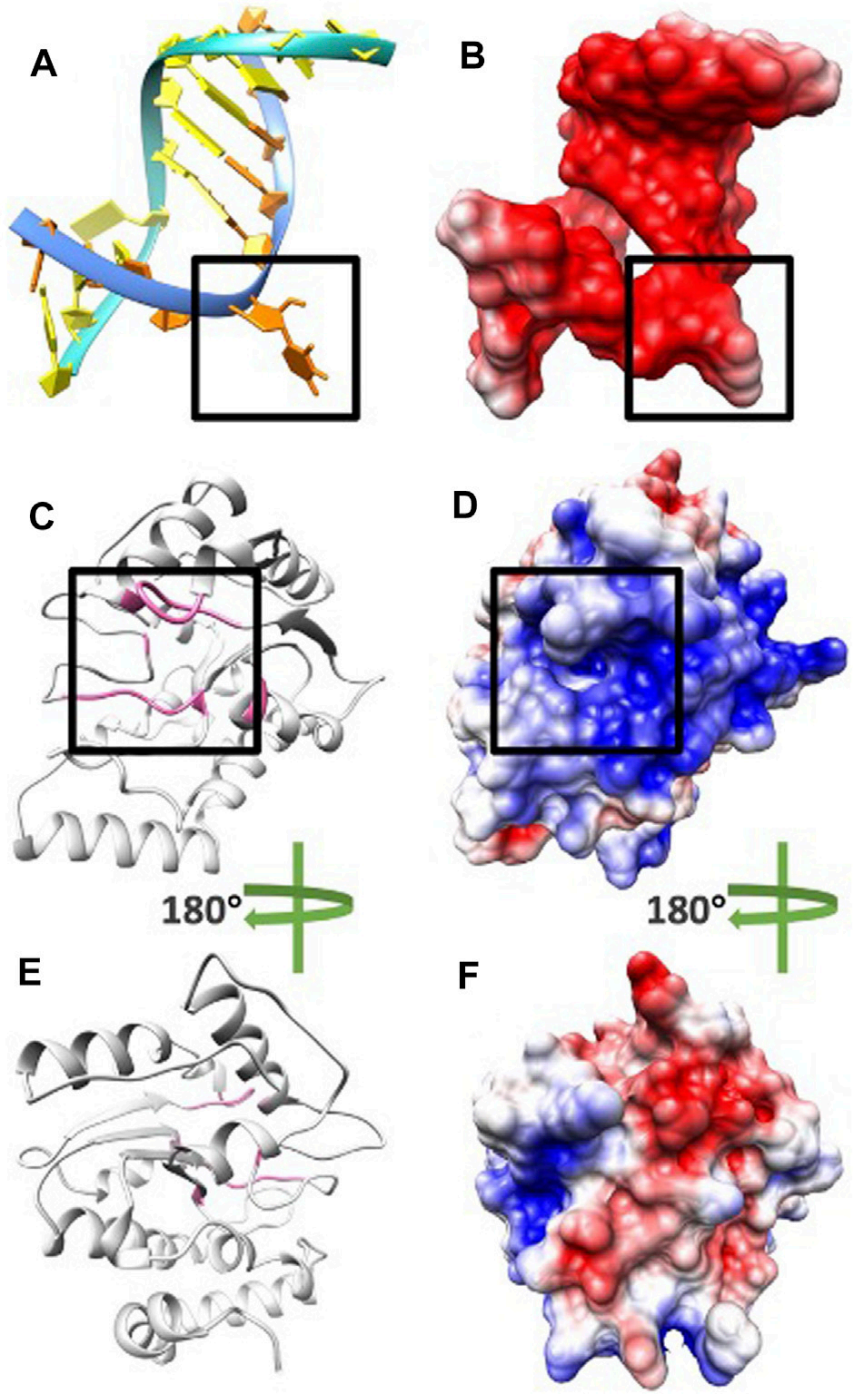
Electrostatic potential on surfaces of DNA and UDG. **(A)** DNA structure orange base at location B5 flips out to the pocket due to UDG mechanism. **(B)** The electrostatic potential on the surface of DNA. **(C)** UDG enzyme structure (pocket side) and the residues of the binding pocket are colored in pink. **(D)** The electrostatic potential on the surface of UDG front side and the black square is the pocket area. **(E)** UDG enzyme structure (non-pocket side) and the residues of the binding pocket are colored in pink. **(F)** The electrostatic potential on the surface of UDG non-pocket side. In figures BDF, the negatively and positively charged areas are colored in red and blue, respectively.

For the calculation parameters in DelPhi, the grid resolution was set to be 2.0 grids/Å. The dielectric constants were set as 2.0 for protein and 80.0 for the water environment. The probe radius for generating the molecular surface was 1.4 Å. The salt concentration was 0.15 M. The boundary condition for PBE was set as a dipolar boundary condition. After the calculation, the values of electrostatic potential on the surface were visualized with Chimera (Figure 4). In order to visualize the electric field lines between DNA and UDG, the separation distance of the DNA from UDG was set to 20Å with respect the direction of their mass centers connection line. Visual molecular dynamics (VMD) (Humphrey et al., 1996) was implemented based on the electrostatic potential map from DelPhi calculations, and the color scale range was set from −3.0 to 3.0 kT/e. For a better representation of electric field lines, we chose the line size to be 4 and delta value to be 0.25, and set the gradient magnitude value to be 3.64 (which shows the lines within the volumetric). Besides, those selected field lines for display have the minimum line length of 4.12 and maximum line length of 35.31.

### Relative Folding Energy Calculation

The net charges of proteins at the unfolded state were calculated using the following equation:

$$Q_{u}\left(\mathrm{pH}\right)=\overset{N}{\underset{i=1}{\sum }}\frac{10^{-2.3y\left(i\right)\left(\mathrm{pH}-\mathrm{pKa}\left(i\right)\right)}}{1+10^{-2.3y\left(i\right)\left(\mathrm{pH}-\mathrm{pKa}\left(i\right)\right)}}\;,$$ (2)

where the summation is of all the titratable groups, y(i) value is −1 for acidic groups, and +1 for basic groups, respectively. As for the folding free energy, the next equation was implemented:

$$\mathrm{\Delta N}\left(pH_{\mathrm{folding}}\right)=2.3\mathrm{RT}\overset{pH_{f}}{\underset{pH_{i}}{\int }}\left(Q_{f}\left(\mathrm{pH}\right)-Q_{u}\left(\mathrm{pH}\right)d\left(\mathrm{pH}\right)\right),$$ (3)

where $Q_{f}\left(pH\right)$ and $Q_{u}\left(pH\right)$ stand for the net charge of folded and unfolded state, respectively. R is the universal gas constant taken as $1.9872\times 10^{-3}\frac{kcal}{Mol*K}$ and T is the temperature with the value of 300 K.

DelPhiPKa (Wang et al., 2015a; Wang et al., 2015b) was used to calculate the pH dependence of folding energy for UDG, given the pH ranging from 0 to 14 with the pH interval of 0.5. During the calculations, we used the AMBER force field (Wang et al., 2004). Water molecules and HETATM were removed because the implicit solvent model is used in DelPhiPKa. Variance of Gaussian Distribution was set to be 0.7, salt concentration was 0.15 M, reference dielectric was 8.0, and external dielectric was 80.0. Note that this method calculated relative folding energies. The folding energy at pH = 0 was set as reference (0 kcal/mol). Lower folding energy at certain pH value indicates higher stability at that pH.

### Electrostatic Binding Forces Calculation

To compare the strengths and directions of electrostatic forces between DNA and UDG, DelPhiForce (Li et al., 2016b) was implemented to perform the force calculations. During DelPhiForce calculations, grid solution was set to be 2.0 grids/Å and salt concentration was 0.15 M. In order to study the binding process of DNA to UDG, we separated the DNA from UDG in the direction of their mass centers connection line with the distances ranging from 20Å to 40Å with the step size of 4Å.

**FIGURE 5:**
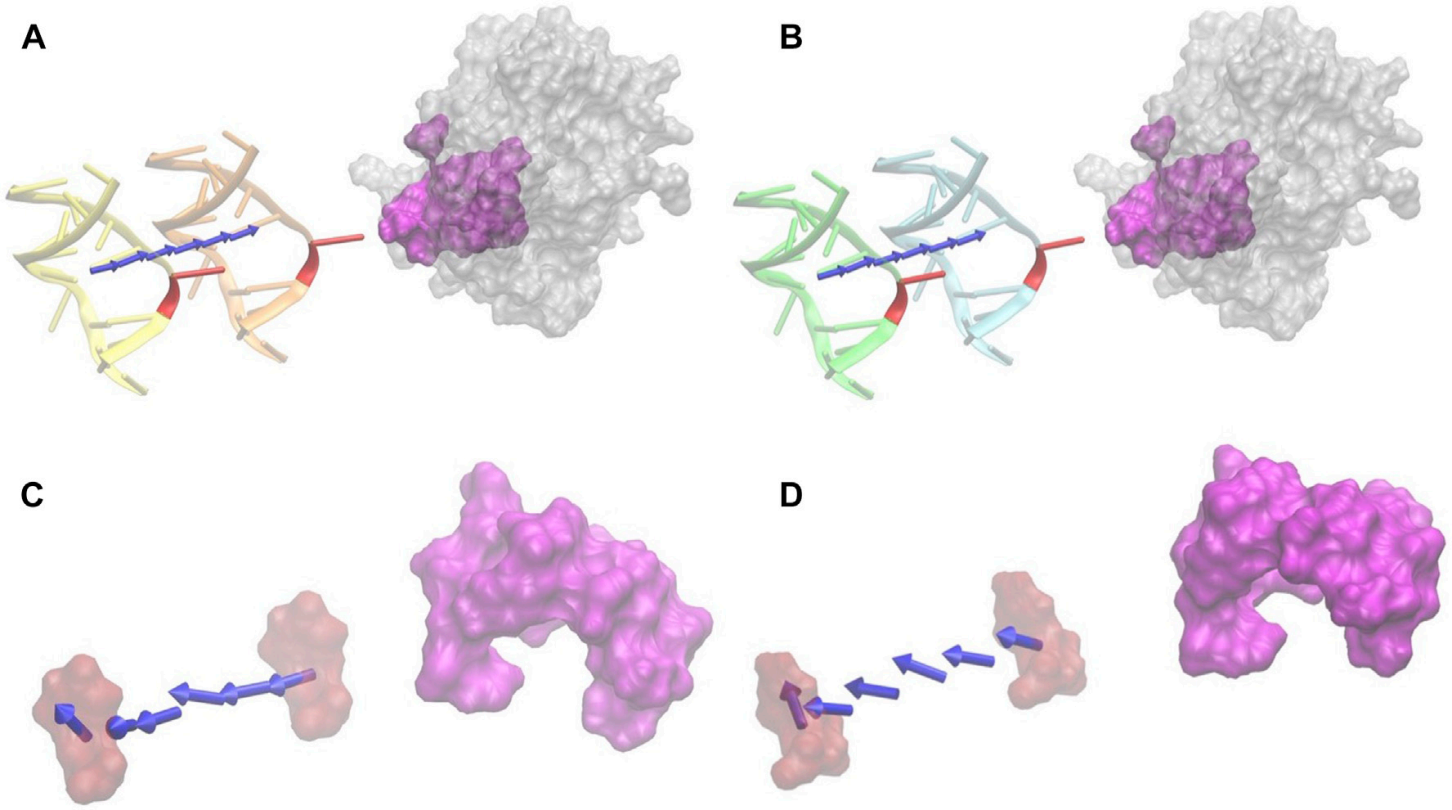
Electrostatic forces of DNA_RU and DNA_C at variable distances with UDG. **(A)** The electrostatic-binding force directions between DNA_RU and UDG with the distances from 20 Å (orange) to 40 Å (yellow) with the step size of 4 Å. Red marks the B5 base uracil in the DNAs, and magenta is the binding pocket area in UDG. **(B)** The electrostatic binding force directions between DNA_C and UDG with the distances from 20 Å (cyan) to 40 Å (green) with the step size of 4 Å. Red marks the B5 base cytosine in the DNAs, and magenta is the binding pocket area in UDG. **(C)** The electrostatic force directions between the uracil base (red) and the UDG pocket (magenta) with the distances from 20 Å to 40 Å. **(D)** The electrostatic binding force directions between cytosine base (red) and the UDG pocket (magenta) with the distances from 20 Å to 40 Å. In this figure, only the force directions are considered rather than the force strengths.

The electrostatic binding forces calculated by DelphiForce were visualized with VMD and represented by arrows. The arrows in Figure 5 represent the directions of forces between DNA and UDG, as they were normalized to be of the same size. In order to study the base B5 (RU or C) and the UDG pocket in particular, apart from the calculations between DNA and the whole UDG, we also did the same calculations of B5 and UDG, as well as B5 and the UDG pocket (Figures 5C,D).

## Results and Discussions

First of all, we analyzed the structures of DNA and UDG, particularly including the UDG binding pocket. Second, the pH dependence of UDG folding energy was calculated and analyzed. Third, the electrostatic features including potential and electric field lines were calculated and investigated. Finally, electrostatic binding forces between DNAs and UDG were analyzed and compared between DNA_RU and DNA_C.

### UDG/DNA Complex Structure

As discussed in the Introduction section, UDG is able to detect the damaged DNA base pair AU in a double-stranded DNA by identifying the unusual kink of 45° (Satange et al., 2018). The uracil base then flips out to the UDG binding pocket so that UDG hydrolyzes the uracil from DNA. Figure 1 shows the binding state of uracil to the UDG pocket.

### pH Dependence of UDG Folding Energy

To better understand the stability of UDG in different environments, especially at different pH values, we calculated the pH dependence of UDG folding energy by using DelPhiPKa.

The calculation was performed at different pH values ranging from 0 to 14 with an interval of 0.5 (Figure 2). From the trends in Figure 2, we observed that the folding energy decreases from 0 to 5, then it becomes relatively more stable from 5 to 10, and increases from 10 to 14. Figure 2 indicates that UDG is stable at pH ranging from 5 to 10. Therefore, the average value of optimal pH is 7.5, which matches the storage conditions of UDG in the laboratory (Wu et al., 2015). Note that the folding energies in Figure 2 are relative values because we set the reference energy to be 0 kcal/mol when pH is equal to 0. We did not calculate the absolute folding energies (energy difference between folded and unfolded states) since we focused on the pH dependency of the folding energies (energy difference between folded energies at a certain pH and pH 0).

### UDG Binding Pocket

DNA always binds to UDG at the pocket side (colored as magenta in Figure 3; sequence: Q144, D145, P146, Y147, H148, F158, S169, S247, H268, P269, S270, P271, L272, and S273) rather than the other side. This is crucial for the binding process since only the pocket area is able to “cut” uracil in DNA instead of other regions on UDG. But the factors to guide DNA binding with the UDG binding pocket efficiently are not fully understood; here, we illustrated the electrostatic potential on the surface of UDG to demonstrate the binding mechanism.

### Electrostatic Potential on Surfaces

To study the electrostatic features, DelPhi was utilized to calculate the electrostatic potential on the surfaces of DNA and UDG. The electrostatic potential distribution on DNA is shown in Figure 4B and movie 1 (see the Supplementary Material), which were rendered by Chimera with a color scale from −3.0 to 3.0 kT/e. The charge distribution on UDG is shown in Figures 4DF and movie 2 (see the Supplementary Material), which were rendered by Chimera with a color scale from −3.0 to 3.0 kT/e as well, negatively and positively charged areas are colored in red and blue, respectively.

By comparing the electrostatic potential on the surfaces of the pocket side and the non-pocket side of UDG, it is clear that UDG has a polar charge distribution. The pocket side has an overall positively charged surface, while the non-pocket side has dominantly negative surface. This charge distribution helps to increase the binding efficiency and decrease the binding direction errors, which ensures the DNA binds directly to the pocket, followed by other processes. Similarly, such polar distributions are commonly found in many other protein–protein interactions, such as molecular motors binding with microtubules (Li et al., 2016b), viral capsid binding with each other (Xian et al., 2019), and enzyme binding with inhibitors (Li et al., 2017b).

Next, by analyzing the electrostatic potential on the surfaces of DNA and UDG, it is obvious that DNA has a negatively charged surface (Figure 4B) while UDG (pocket side; Figure 4D) has a positively charged surface. This fact indicates the attractive forces between DNA and UDG (pocket side). In order to investigate more about the attractive interactions, field lines were generated between DNA and UDG, which is discussed in the Electric field lines section.

**FIGURE 6:**
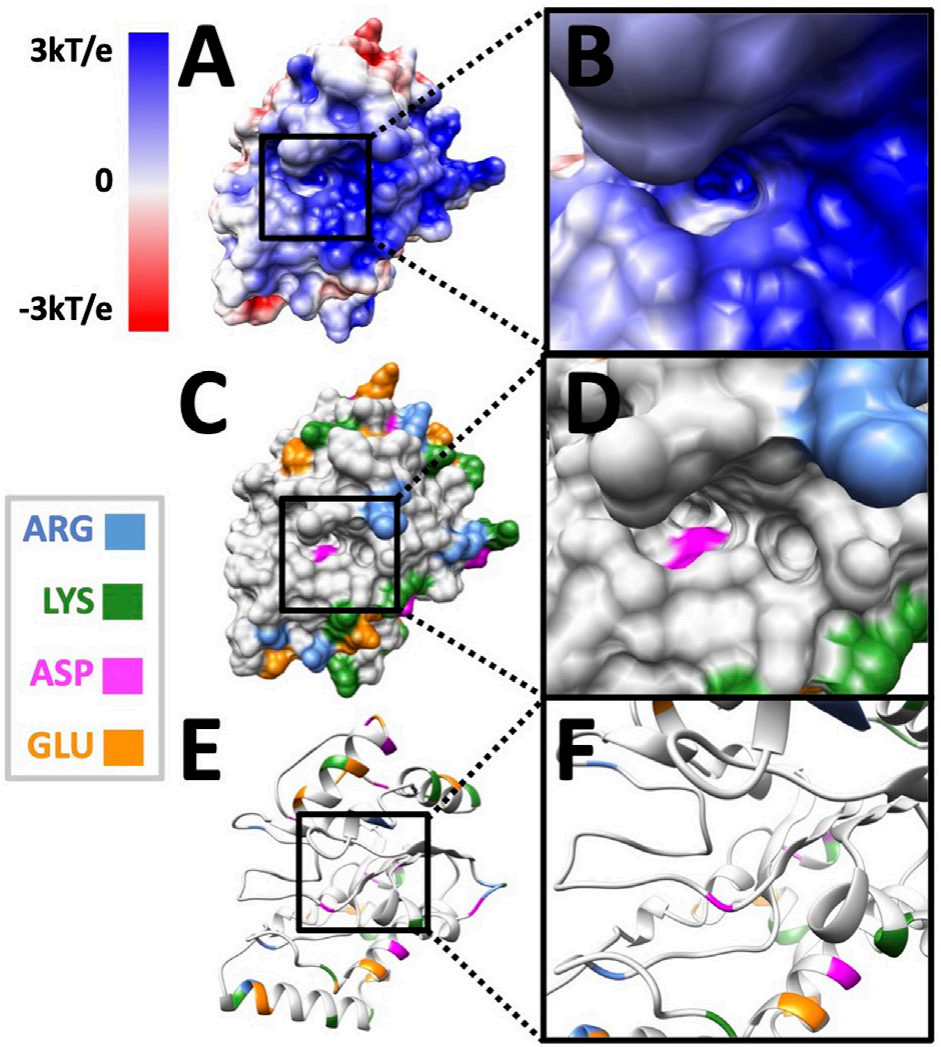
The residue distributions on the surface of UDG. **(A)** The electrostatic potential on the surface of the UDG pocket side. **(B)** The electrostatic potential on the surface of the UDG binding interface area. **(C)** The surface of UDG with the colored charged residues as shown in the legend. **(D)** The surface of the UDG binding interface area. **(E)** The structure of UDG with the colored charged residues. **(F)** The structure of the UDG binding interface area.

After looking at the overall potential distribution, we colored charged amino acids in Figures 6C,F to visualize the charge distribution.

In Figure 6, black squares indicate the area of binding interface of UDG when DNA binds to it. By looking at Figure 6B, the binding interface is overall blue, which is positively charged. While by looking at Figure 6D, there is only one negatively charged residue (ASP in pink) inside the pocket, while the surrounding area of the binding interface has several positively charged residues (ARG in blue and LYS in green). This fact indicates that the binding pocket itself does not provide the attractive forces to DNA, since DNA is overall negatively charged. However, the whole binding interface does provide the positively charged environment for DNA to be attracted and bound to UDG.

In order to investigate more about the pocket area in particular, we calculated the forces between DNA and UDG with different partial complex structures that are DNA/UDG, DNA (B5 base only)/UDG, and DNA (B5 base only)/UDG (pocket only). Those results are discussed in the Electronic forces section.

### Electric Filed Lines

**FIGURE 7:**
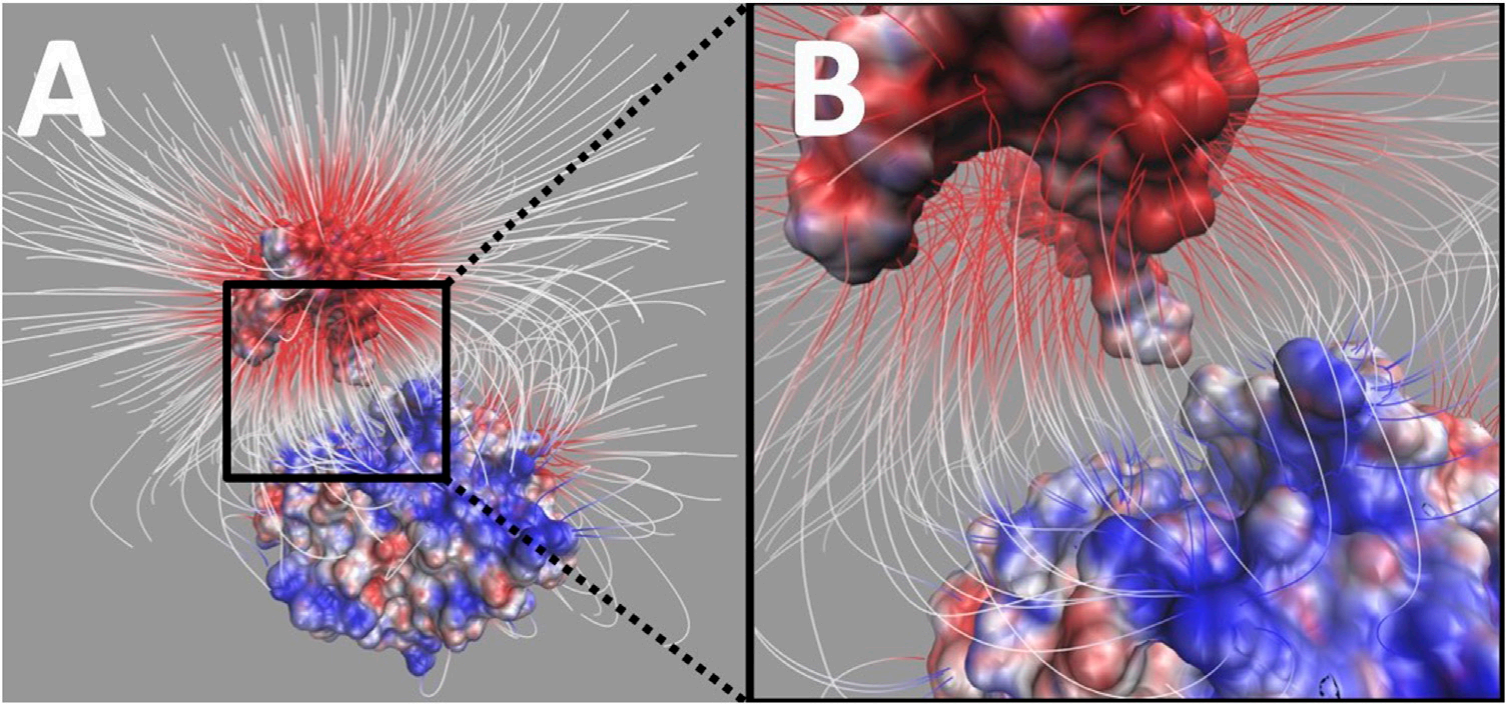
Electrostatic filed lines between DNA and UDG. **(A)** The overall view of electrostatic filed lines distribution between DNA (top) and UDG (bottom). **(B)** A closeup of electrostatic field lines distribution between DNA (top) and UDG (bottom) at the interface area. Visual molecular dynamics (VMD) (Kortemme et al., 2004) was implemented based on the electrostatic potential map from DelPhi calculations, and the color scale range was set from −3.0 to 3.0 kT/e. For the better representation of electric field lines, we chose the line size to be four and delta value to be 0.25, and set gradient magnitude value 3.64 (which shows the lines within the volumetric). Besides, those selected field lines for display have the minimum line length of 4.12 and maximum line length of 35.31.

Electric field lines between UDG and DNA were calculated. To better visualize the field lines between the binding interface, DNA was separated from UDG by 20 Å (Figure 7).

The field lines distribution confirmed that UDG and DNA have attractive forces between each other. In the analysis of field lines, the density of the distribution indicates the strength of the electrostatic binding forces, which means that the denser distribution has the stronger interactions. From Figure 7B, we noticed that the flipping out base uracil has a very dense field lines connected to the UDG binding pocket.

### Electrostatic Forces

**FIGURE 8:**
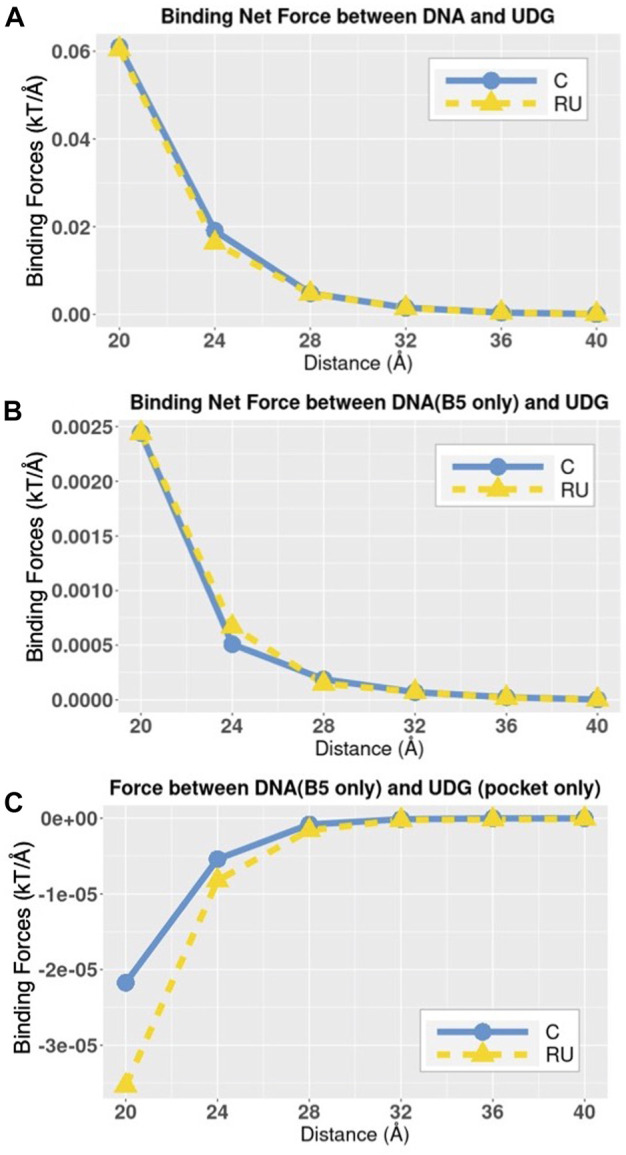
The trends of total electrostatic forces between DNAs and UDG. **(A)** Electrostatic-binding net forces between DNA and UDG at distances from 20 Å to 40 Å with the step size of 4 Å. **(B)** Electrostatic-binding net forces between DNA (B5 base only) and UDG at distances from 20 Å to 40 Å with the step size of 4 Å; in DNA_RU the B5 base is uracil and in DNA_C the B5 base is cytosine. **(C)** Electrostatic-binding net forces between DNA (B5 base only) and the UDG pocket at distances from 20 Å to 40 Å with the step size of 4 Å; in DNA_RU the B5 base is uracil and in DNA_C the B5 base is cytosine.

Electrostatic forces of DNA and UDG were calculated by DelPhiForce. The calculated results were visualized with arrows (Figure 5) and line graphs (Figure 8). Arrows in Figure 5 represent the net forces between DNA and UDG by shifting the DNA away from UDG by variable distances ranging from 20 Å to 40 Å with the step size of 4 Å. The direction of arrows represents the force directions. To better visualize the direction of the net forces, the magnitudes of the net forces were normalized to be of the same size, which means that the size of the force does not represent the force strength. Force strengths are discussed in the Force strengths section and visualized in Figure 8.

#### Force Directions

From Figures 5A,B, it is obvious that UDG has attractive forces to DNA at distances ranging from 20 Å to 40 Å, no matter the B5 base in DNA is uracil (Figure 5A) or cytosine (Figure 5B). While by looking at Figures 5C,D, the UDG pocket provide repulsive forces to DNA B5 base at distances ranging from 20 Å to 40 Å, no matter the B5 base in DNA is uracil (Figure 5C) or cytosine (Figure 5D). This fact verifies the previous conclusion in the Electrostatic potential on surfaces section that the UDG pocket alone does not provide the binding attractive force to DNA but that the whole binding interface provides the binding attractive force to the DNA.

#### Force Strengths

In the Force directions section, we discussed the force directions rather than force strengths. In this section, we discuss the force strengths as well as the comparison between DNA_RU and DNA_C in three different complex structures.

In order to know the total electrostatic binding net forces between DNA and UDG at variable distances, we calculated the force strengths between whole DNA and the whole UDG (Figure 8A). Since B5 base is the one that is different between DNA_RU and DNA_C, we also calculated the force strengths between DNA B5 base alone and the whole UDG (Figure 8B). Besides, in order to analyze the DNA B5 base and the UDG binding pocket in particular, we calculated the force strengths between these two components (Figure 8C).

From the trends in Figure 8, the electrostatic binding forces, no matter attractive or repulsive, decrease along with the increasing distances. This fact shows that the electrostatic forces between DNA and UDG are not the specific forces to distinguish DNA_C and DNA_RU. By looking at Figure 8C in particular, it again shows that the B5 base and the UDG binding pocket have repulsive forces between each other, so it is the UDG binding interface rather than the pocket that attracts DNA. We also found that compared to the cytosine base, the uracil base generally forms stronger attractive forces to UDG (Figure 8B) and stronger repulsive forces to the UDG pocket (Figure 8C), but the differences between the force strengths generated by uracil and cytosine are insignificant. C and RU bases have the same net charge, which is −1 e. The electrostatic force differences were resulted from the charge distributions in C and RU bases. Therefore, the insignificant force difference is reasonable.

The limitation for this study is that we calculated relative folding energies rather than absolute energies. Since our study is focused on the stability under the pH effects, the use of relative folding energy calculation is necessary to get our conclusions. Another limitation is that we only considered electrostatic interactions in this study. Taking into account more interactions such as van der Waals forces, hydrogen bonds, and salt bridges will provide more comprehensive perspective to understand the interactions between biomolecules (Li et al., 2015). Therefore, we plan to study the other interactions in our future study.

## Conclusion

DNA damage occurs in every cell all the time and may lead to unpredicted consequences to human health. DNA repair is an essential process as it provides comprehensive coverage of cellular responses to DNA damage. Uracil-DNA glycosylase (UDG) is one of the most important enzymes in base excision repair (BER), one of the DNA repair mechanisms. During its repairing process, UDG first scans the DNA backbone for the uracil base, then uses its “pocket” to closely bind to the uracil, and finally “cuts” this uracil.

In this study, with the help of multiple computational approaches: DelPhiPKa for the pKa calculation; DelPhi and DelPhiForce for the electrostatic feature calculations; and data analysis and visualization with the help of Chimera, VMD, and R language. We analyzed the pH dependency of UDG folding energy and the result shows that UDG achieves the most stable configuration at pH ranging from 5 to 10. Then we calculated the electrostatic potential on the surface of both UDG and DNA, and the analyses of the different charge distributions of the UDG pocket side and the non-pocket side were performed. Moreover, we calculated the electrostatic forces between UDG and DNA, especially the pocket area and target uracil base in DNA. The results demonstrate that UDG has overall attractive forces to DNA at different distances ranging from 20 Å to 40 Å, while the UDG pocket has repulsive forces to the uracil base at the same distance range. Furthermore, the resides in both the pocket area and the interfacial area between UDG and DNA were discussed in detail, which explains the interesting differences of force features between the whole UDG interface and the UDG pocket.

This research provides the essential explanations of the binding mechanisms of UDG and DNA, i.e., the whole UDG binding interface, and not the UDG pocket area alone, provides the binding attractive forces to the damaged DNA. Our study provides a better understanding of DNA repair mechanisms, which may lead to novel UDG enzyme applications.

## Data Availability

The original contributions presented in the study are included in the article/Supplementary Material; further inquiries can be directed to the corresponding author.
