# Supplementary material for: Computational Study on DNA Repair: The Roles of Electrostatic Interactions Between Uracil-DNA Glycosylase (UDG) and DNA
Source: Front Mol Biosci. 2021 Aug 6;8:718587. doi: 10.3389/fmolb.2021.718587 (PMC8377759; doi:10.3389/fmolb.2021.718587)
Supplement: Supplementary file 1 [file DataSheet1.pdf]

Computational Study on DNA Repair: The Roles of Electrostatic Interactions Between Uracil-DNA Glycosylase (UDG) and DNA

Yixin Xie[1], Chitra B. Karki[1], Jiawei Chen[2], Dongfang Liu[3], Lin Li[1,4*]

[1] Computational Science Program, University of Texas at El Paso, El Paso, TX

[2] Computer Science Program, Santa Monica College, Santa Monica, CA

[3] Department of Computer Engineering, Rochester Institute of Technology, Rochester, NY

[4] Department of Physics, University of Texas at El Paso, El Paso, TX

***Correspondence:**
Lin Li: lli5@utep.edu

1 Sequences of UDG (chain A) and DNA (chain B and chain C):

>1EMH_3|Chain A |URACIL-DNA GLYCOSYLASE | Homo sapiens (9606)
MEFFGESWKKHLSGEFGKPYFIKLMGFVAEERKHYTVYPPPHQVFTWTQMCDIKDVKV
VILGQDPYHGPNQAHGLCFSVQRPVPPPPSLENIYKELSTDIEDFVHPGHGDLSGWAKQG
VLLLNAVLTVRAHQANSHKERGWEQFTDAVVSWLNQNSNGLVFLLWGSYAQKKGSAI
DRKRHHVLQTAHPSPLSVYRGFFGCRHFSKTNELLQKSGKKPIDWKEL
>1EMH_1|Chain B |DNA (5'-D(*TP*GP*TP*(P2U) *AP*TP*CP*TP*T)-3') |
TGTUATCTT
>1EMH_2|Chain C |DNA (5'-D(*AP*AP*AP*GP*AP*TP*AP*AP*CP*A)-3') |
AAAGATAACA

2 Movies

Movie1: Electrostatic potential on surfaces of DNA
Movie2: Electrostatic potential on surfaces of UDG
